# Evaluation of Clay Hydration and Swelling Inhibition Using Quaternary Ammonium Dicationic Surfactant with Phenyl Linker

**DOI:** 10.3390/molecules25184333

**Published:** 2020-09-22

**Authors:** Mobeen Murtaza, Hafiz Mudaser Ahmad, Muhammad Shahzad Kamal, Syed Muhammad Shakil Hussain, Mohamed Mahmoud, Shirish Patil

**Affiliations:** 1 Department of Petroleum Engineering, King Fahd University of Petroleum & Minerals, Dhahran 31261, Saudi Arabia; mobeen@kfupm.edu.sa (M.M.); patil@kfupm.edu.sa (S.P.); 2 Department of Chemical, Polymer & Composite Materials Engineering, University of Engineering & Technology, New Campus, Lahore 54890, Pakistan; h.m.ahmad@uet.edu.pk; 3 Center for Integrative Petroleum Research (CIPR), King Fahd University of Petroleum & Minerals, Dhahran 31261, Saudi Arabia; shahzadmalik@kfupm.edu.sa

**Keywords:** quaternary ammonium, gemini surfactants, shale inhibition, drilling fluids, wettability alteration

## Abstract

Water-based drilling fluids are extensively used for drilling oil and gas wells. However, water-based muds cause clay swelling, which severely affects the stability of wellbore. Due to two adsorption positions, it is expected that cationic gemini surfactants can reduce the clay swelling. In this work, quaternary ammonium dicationic gemini surfactants containing phenyl linkers and different counterions (Cl^−^ and Br^−^) were synthesized, and the effect of variation in counterions on swelling and hydration properties of shales was studied. Numerous water-based drilling fluid formulations were prepared with different concentrations of surfactants to study the swelling inhibition capacity of surfactants. The performance of surfactant-containing drilling muds was evaluated by comparing them with base drilling mud, and sodium silicate drilling mud. Various experimental techniques were employed to study drilling mud characteristics such as rheology and filtration. The inhibition properties of drilling mud formulations were determined by linear swelling experiment, capillary suction time test, particle size distribution measurement, wettability measurements, and X-ray Diffraction (XRD). Experimental results showed that surfactant-based formulation containing bromide counterion exhibited superior rheological properties as compared to other investigated formulations. The filtration test showed that the gemini surfactant with chloride counterion had higher filtrate loss compared to all other formulations. The bentonite swelling was significantly reduced with increasing the concentration of dicationic surfactants as inhibitors, and maximum reduction in the linear swelling rate was observed by using a formulation containing surfactant with chloride counterion. The lowest capillary suction timer (CST) was obtained in the formulation containing surfactant with chloride counterion as less CST indicated the enhanced inhibition capacity. The particle size measurement showed that average bentonite particle size increased upon the addition of surfactants depicting the inhibition capacity. The increase in basal spacing obtained from XRD analysis showed the intercalation of gemini surfactants in interlayers of bentonite. The contact angle measurements were performed to study the wettability of the bentonite film surface, and the results showed that hydrophobicity increased by incorporating the surfactants to the drilling fluid.

## 1. Introduction

The growing demand for fossil fuels due to the increased population has shifted the paradigm of the oil and gas industry to develop unconventional reservoirs [1,2,3]. In recent decades, the extraction of oil and gas from shale reservoirs has increased in order to meet the current and future expected demand for oil and gas resources [4]. Shale reservoir formations mainly comprise of water-sensitive clay minerals such as smectite, illite, montmorillonite, and kaolinite [5,6]. Shale reservoir drilling operations often result in complicated wellbore instability problems due to the swelling of sensitive clay minerals present in the shale formations. The wellbore instabilities include bit balling, caving, sloughing, stuck pipe, and in severe cases, the collapse of the wellbore may occur. Therefore, the design and selection of drilling fluid play a significant role in minimizing the wellbore instability problems [7,8,9,10].

Drilling of shale reservoir requires specially designed drilling fluids with enhanced inhibition properties to minimize the shale swelling and hydration. Therefore, the choice of drilling fluids mainly depends on the cost of drilling fluid, environmental challenges, and shale inhibition characteristics [11,12,13,14]. It is reported in the literature that oil-based mud (OBM) has exceptional shale inhibition characteristics, better lubricity and high temperature stability, preserves the integrity of wellbore formations, and is preferred for formations containing water-sensitive shale [15]. The OBM is not frequently employed due to the high cost and stringent environmental regulations [16,17,18]. On the other hand, water-based mud (WBM) provides excellent rheological properties, has low preparation cost, and is environmentally friendly compared to the OBM [19,20]. However, the water contents in the WBM have a high affinity towards the water-sensitive shale in the wellbore formations, which leads to the hydration and swelling of shale [21,22]. Various additives (shale inhibitors) were employed in the formulation of drilling fluids to modify the shale swelling and hydration properties [23,24].

Several drilling fluids additives were employed as shale inhibitors from the last few decades to enhance drilling fluid inhibition properties. The reported shale inhibitors in the literature include polymers [25,26,27]; inorganic slats [28]; polymer nanocomposites [29,30]; glycols [31,32]; amines [33,34] and its derivatives, polymeric amines, dendrimers of amines [35,36], ionic liquids [37], nanoparticles [38], surface-modified nanoparticles [39], and surfactants [3]. The shale inhibition phenomenon is categorized into two classes, inhibition by physically sealing the micropores and small cracks, and chemical inhibition, in which inhibitor molecules encapsulate the shale surface to prevent the interaction of shale with water. The inhibitors that follow the chemical inhibition are considered as superior compared to the inhibitors who follow the physical sealing mechanism. There are some limitations to the applications of the additives mentioned above. For instance, potassium chloride has enhanced inhibition capacity, but it cannot be used with high concentration due to high toxicity to the marine environment. It also stimulates the dispersion of kaolin clay and results in bit balling [40,41]. Polymers and their composites as shale swelling inhibitors are not effective at high temperature conditions encountered in the deep wells [42,43]. The amine-based shale swelling inhibitors have limited applications due to some associated disadvantages that include toxicity, pH-dependent inhibition, high dosage, and low temperature tolerance [44,45,46]. For nanoparticles, their homogenous distribution in drilling fluids and high impact on rheological properties are the main concern in their application [40,47]. Considering the limitations of current solutions, developing a novel shale swelling inhibitor with terrific inhibition performance is still a research hotspot. Therefore, a new class of chemical inhibitors such as surfactants are introduced, which can play a significant role in developing drilling fluids with high inhibition properties.

Several studies have been conducted to investigate the potential of surfactants as a shale swelling inhibitor [48,49]. It was noticed that surfactants showed promising potential as a swelling inhibitor by modifying the surface properties of the clay. There was little focus on the potential use of gemini cationic surfactants as a shale inhibitor [50,51]. Gemini surfactants are a class of surfactant that contains more than one head and tail group connected with a spacer [52,53,54,55,56]. Gemini surfactants have recently been employed for many applications in the oil and gas industry due to their unique properties such as lower critical micelle concentration (CMC), higher interface/surface properties, excellent solubility, high thermal stability, and unique aggregation behavior as compared to their monomeric counterparts [57,58,59,60,61]. Gemini cationic surfactants have gained much attention because they have two adsorption positions compared to conventional surfactants. As a result, gemini surfactants can enhance their adsorption on clay surface due to their higher cation exchange capacity [62]. Although quaternary ammonium compounds are also toxic to the marine environment, dimeric gemini surfactants have low toxicity, which can be further reduced by adding a hydrophilic group [63]. Gemini surfactants used in this study possess two long-chain hydrophobic chains along with ethoxy groups attached with two quaternary ammonium heads, and the quaternary ammonium heads are connected through a spacer or linker, which impart the distinctive characteristics to the gemini surfactants [64]. The quaternary ammonium ions chemically interact with the clay minerals present in the shale, and long-chain hydrophobic groups encapsulate the shale surface protecting the interaction with water [50,51,60,65,66,67]. Gao et al. used the novel gemini surfactant 2,2′-bis(dodecyldimethylammonio)-ethyl ether dichloride to prepare the organophilic clay [68]. Ren et al. used the novel gemini surfactants to make the organophilic clay, and the effect of alkyl chain length on adsorption properties was determined by the methyl orange test [69]. The functional mechanism of surfactants to change the shale surface from hydrophilic to hydrophobic was analyzed by using cetyl trimethyl ammonium bromide (CTAB), and resultantly, the surfactant improved the wettability of shale surface [70].

In this work, two gemini surfactants containing chloride and bromide counterions (**PC** and **PB**) were synthesized in the laboratory and the effect of chloride and bromide counterions on rheology, filtration and shale-inhibition properties were investigated. The influence of concentration of surfactant on rheological properties was studied and compared with base and sodium silicate containing drilling fluids. The filtration characteristics were assessed by the filter press apparatus. The shale inhibition characteristics of gemini surfactant that contained drilling fluids were determined by performing linear swelling and capillary suction timer tests. The effect of synthesized surfactant on coagulation of bentonite particles was determined by performing particle size measurements. The wettability alteration of the shale surface was analyzed by contact angle measurements. The change in basal spacing was investigated by XRD method.

## 2. Results and Discussion

The cationic gemini surfactants have two adsorption positions and also have high cation exchange capacity. However, most of the cationic gemini surfactants have poor salt tolerance and can precipitate in the presence of divalent cations [60,71]. These surfactants were designed to tolerate the typical high temperature and high salinity oilfield conditions. This section discusses how the addition of a surfactant impacts rheological properties, filtration properties, and clay swelling inhibition.

### 2.1. Rheological Properties

The rheological properties of drilling fluids play an essential role in the smooth drilling operation. The effect of the surfactant nature on the rheological properties of drilling fluids was determined using a rotational viscometer. The designing of drilling fluid properties with appropriate drilling fluid additives is significant to control its effect on drilled cutting transport, cutting suspension capacity, and circulation of fluid around the pipes in the wellbore.

Table 1 shows the apparent viscosities (AVs) of various drilling mud samples. In the Bingham plastic rheology model, the AV was determined to assess the flowability of prepared drilling mud at a fixed shear rate. The AV of base mud (BM) was 11.06 cp and was observed as the highest value as compared to the rest of the formulations. For sodium silicate mixed mud, it was noticed that the AV was reduced to 7 cp upon its addition in the base mud. The addition of 0.1% gemini surfactant (**PC** and **PB**) to the base drilling mud also reduced AV as compared to the base mud. The drilling mud formulation containing 0.1% **PC** shows AV of 7.44 cp, while the drilling mud formulation having 0.1% **PB** exhibits 8.32 cp as AV.

Table 1 provides the results of another critical rheological property called plastic viscosity (PV) of drilling muds, measured at room temperature. The plastic viscosity has a direct relation with the solid contents of drilling muds and the temperature at which the viscosity was measured. It plays a vital role in the carrying capacity of drilling muds. In this study, the temperature was kept constant to study the effect of **PC** and **PB** on the PV of drilling muds. First, the base mud (BM) showed the most significant value of 8.22 cp PV as compared to all other formulations. Second, the addition of 0.1% inhibitors to base mud resulted in the reduction of PV. The formulation with 0.1% of sodium silicate (SS) provided a PV of 6.16 cp. On the other hand, the formulations with 0.1% of **PC** and **PB** resulted in 5.58 cp and 5.97 cp as plastic viscosities, respectively.

Yield point (YP) of drilling mud implies the ability to transport the drilled cuttings from the subsurface to the surface. Drilling mud that has a high value of YP can transport drilled cuttings to the surface better compared to drilling mud that has a low value of YP. On the other hand, the extremely high value of YP of drilling muds requires high pumping power that leads to high operational costs. Table 1 shows the values of the YP of various drilling mud formulations measured at room temperature. The base mud showed the maximum value of YP of 5.67 (l bf/100 ft^2^) compared to the rest of the drilling mud formulations. The drilling mud formulation with sodium silicate as a shale inhibitor (SS) showed a 72% reduction in YP compared to the BM. The addition of **PC** and **PB** also affected the YP of formulations, but their effect was less prominent compared to the (SS)-based formulation. The **PC**- and **PB**-based formulations showed a 34% and 17% reduction in YP, respectively, compared to the BM.

**Figure 1:**
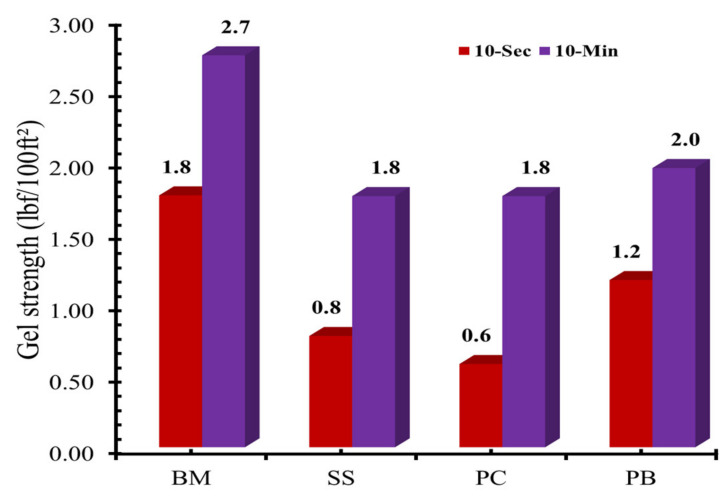
The gel strength of base mud (BM), 0.1% of SS mud, and modified mud with 0.1% of **PC** and **PB**.

The gel strength of drilling mud formulation determines the inter-particle interaction and gelling capacity of drilling mud additive after the rotation of the drill bit stopped. Gel strength also signifies the importance of drilled suspension capacity of drilling mud during the static period. Figure 1 shows the gel strength of various drilling mud formulations measured at room temperature. The maximum value of gel strength was observed for BM, while the gel strengths of all other formulations were affected by the addition of sodium silicate as well as **PC** and **PB**. The 10-s and 10-min gel strengths of SS formulation were reduced by 55% and 33%, respectively, compared to the BM. The **PC**-based formulation shows 66% and 33% reduction in 10-s and 10-min gel strengths. The **PB**-based formulations exhibit 33% and 26% reduction in 10-s and 10-min gel strengths.

The overall impact of **PC** and **PB** on rheological properties shows that the incorporation of surfactant reduces the rheological properties. The reduction in rheological properties is attributed to the breakage of the three-dimensional card house structure in the base mud [49,72]. The addition of surfactants leads to an agglomeration of bentonite particles, which occurs upon reduction in repulsive forces of bentonite particles in the drilling mud. The well-dispersed clay particles in BM moved to aggregation upon the addition of a surfactant [73,74]. The presence of two ammonium cations in the **PC** and **PB** molecules plays a vital role in the reorientation of inter-particle interactions. The ammonium cations interact electrostatically with the negatively charged clay surface and make strong bonds.

Further, the two long chains of each surfactant molecule also disrupt the inter-particles interaction among clay platelets. The long chains in **PC** and **PB** molecules and the interaction of ammonium ions with clay lead to the reduction in rheological properties. Ideally, the swelling inhibitors and clay stabilizers should not affect the rheological properties of drilling mud. However, in the case of clay-based drilling fluids, it is reported in the literature that natural surfactants reduce plastic viscosity and yield point of clay-based drilling mud by preventing dispersion of clay [48]. Similarly, most of the commercial inhibitors like sodium silicate and KCl affect the rheological properties of the clay-based drilling mud [75,76]. However, the impact of the gemini surfactant on the rheological properties of bentonite-based drilling mud was less compared to the sodium silicate.

### 2.2. Filtration Properties

**Figure 2:**
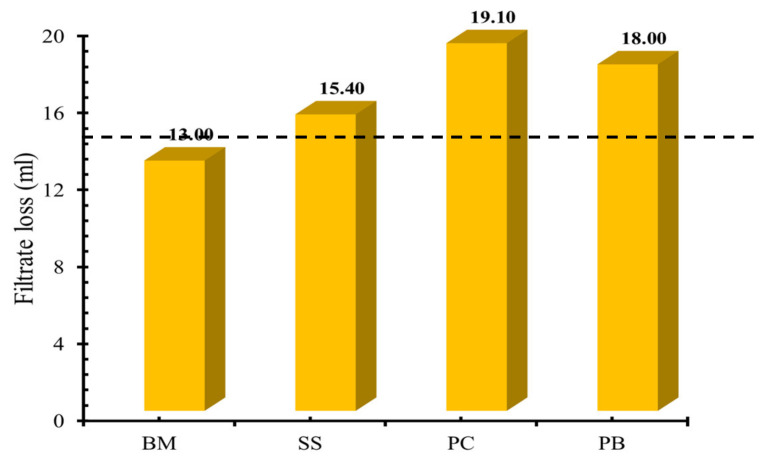
Filtrate loss of base mud (BM), 0.1% of SS mud and modified mud with 0.1% of **PC** and **PB**.

Filtration volume and filter cake thickness are critical parameters to analyze the performance of drilling mud formulation. Less filtrate volume indicates better preservation of wellbore formations and less contamination of drilling mud with formation mud. Figure 2 shows the effect of different shale inhibitors on filtrate volume for various drilling mud formulations. The BM formulation provided 13 mL of filtrate loss while the addition of sodium silicate increased the filtrate volume to 15.4 mL. It was observed that the addition of gemini surfactants increased the filtrate volume of bentonite-based drilling muds. The drilling mud formulations containing **PC** and **PB** resulted in a filtrate volume of 19.10 mL and 18 mL, respectively. The increase in fluid loss depicts the suppressing of bentonite swelling by the tested surfactants as they impede the bentonite hydration by reducing its water affinity. Similar phenomena were observed in applications of other inhibitors in clay-based drilling fluids [49,77].

**Figure 3:**
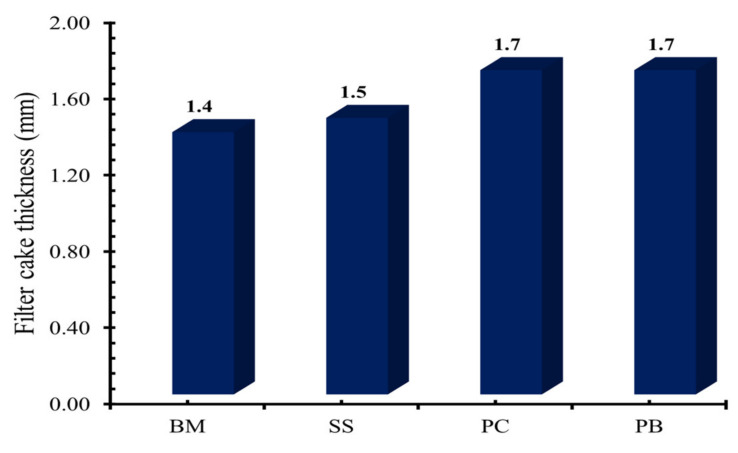
Filter cake thickness of base mud (BM), 0.1% of SS mud, and modified mud with 0.1% of **PC** and **PB**.

Figure 3 shows the filter cake thicknesses measured after performing the filtration experiment. The BM formulation showed a minimum thickness of filter cake (1.4 mm), which is lowest compared to all other filter cakes. The addition of sodium silicate and **PC** and **PB** to the base mud increased the filter cake thickness. The filter cake of SS formulation increased by 7%, while the filter cake thickness of **PC** and **PB** formulations increased by 21.5% as compared to the filter cake thickness of BM formulation.

The overall results of filtration analysis show that fluid loss volume increases with the addition of gemini surfactants. The increased fluid loss and filter cake thickness of **PC** and **PB** formulation can be explained by the intercalation of surfactant molecules into the layers of clay. The increase in the filtrate loss was also observed with the inclusion of KCl as a shale inhibitor in the drilling mud, as reported by Mobeen et al. [51]. The filtrate loss volume of less than 15 mL is considered favorable for a smooth drilling operation. However, the addition of shale inhibitors in the bentonite-based drilling mud slightly increased the amount of filtrate loss described by API standards [77]. Therefore, the formulations having gemini surfactants show a higher fluid loss and filter cake thickness. It is common practice to add the fluid loss controllers such as starch and polyanionic cellulose (PAC) in the drilling mud containing shale-swelling inhibitors. These fluid loss controllers prevent the filtrate loss of water into the formation and maintain the drilling fluid properties [78].

**Figure 4:**
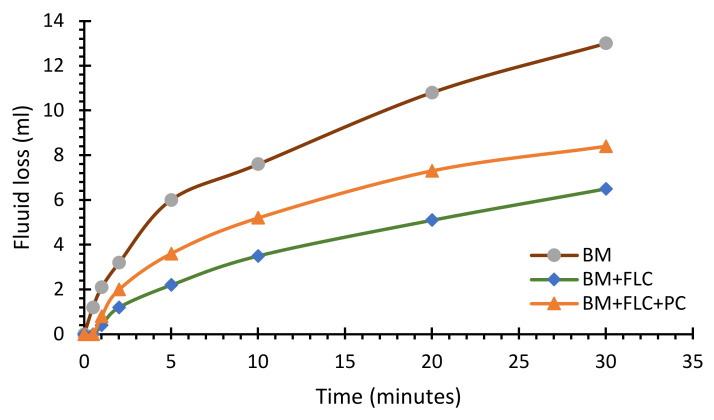
The filtration test on three different formulations (BM, BM + FLC and BM + FLC + 0.1% **PC**).

Also, a filtration test was conducted on formulations containing starch as a fluid loss controller (FLC) and **PC** as a surfactant to investigate its performance in the presence of FLC. The starch was mixed by 3 g in the 350 mL of BM and conducted the filtration test as the results are given in Figure 4. It was observed that the addition of FLC reduced the filtrate loss of BM from 13 mL to 6.5 mL at the end of 30 min. Further, the **PC** surfactant performance was evaluated in the presence of FLC. The **PC** was mixed with 0.1 wt% concentration in the mud containing FLC. The filtrate loss was increased slightly from 6.5 mL to 8.4 mL, which was quite a lot less compared to the BM. So, it is pertinent to add the FLC in the clay-based drilling mud formulations containing gemini surfactant.

### 2.3. Contact Angle Measurements

**Figure 5:**
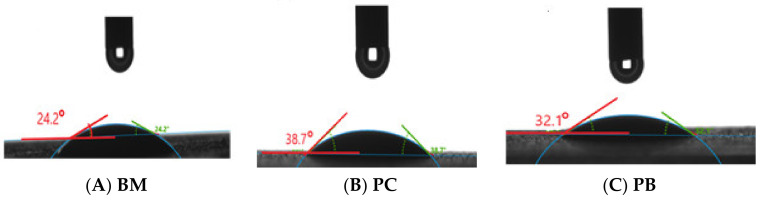
Contact angle measurement (**A**) Base mud (BM), (**B**) Drilling mud with 0.1% concentration of **PC,** (**C**) Drilling mud with 0.1% concentration of **PB**.

The contact angle measurement provides an indication of the wettability of treated and untreated surfaces in terms of hydrophilic and hydrophobic surface. Figure 5 shows the contact angles of base mud and gemini surfactant mixed muds. The base mud (BM) without shale inhibitor shows the approximate contact angle 24.2°. The incorporation of **PC** and **PB** increased the contact angles to 38.7° and 32.1°, respectively.

The measurement of the contact angle of the film surface modified with gemini surfactant (**PC** and **PB**) proved that the addition of gemini surfactant to water-based drilling muds increased the hydrophobicity of the clay surface [69,79]. The gemini surfactant molecules consist of two quaternary ammonium cations, a benzene ring as a spacer, and two long chains of alkyl groups. The spacer benzene ring modifies the wettability of the film surface, and long-chain alkyl groups make a thin hydrophobic layer that prevents the water penetration into the shale or clay formation.

### 2.4. Linear Swelling

**Figure 6:**
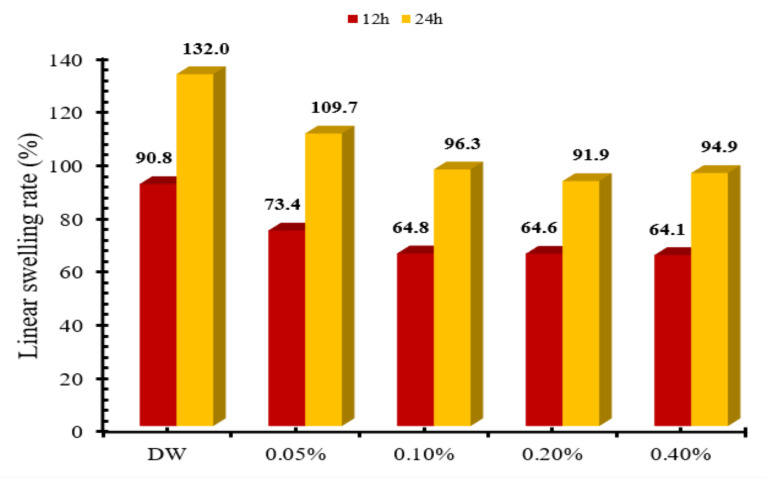
Effect of **PC** concentration and time on the linear swelling rate.

The linear swelling test indicates the inhibition characteristics of shale inhibitors (**PC** and **PB**) used in this study. The bentonite pellet was employed to analyze the impact on inhibition by measuring the linear vertical height of pellet or linear swelling rate (%) for different time intervals. The effect of **PC** and **PB** on the linear swelling rate of bentonite pellet was analyzed for 12 h and 24 h. Figure 6 shows the impact of **PC** concentration on the linear swelling rate for 12 h and 24 h. It was observed that the swelling rate of bentonite was maximum in the deionized water due to the direct hydration of pellets in the presence of deionized water. The addition of **PC** reduced the linear swelling rate for both 12-h and 24-h periods. The increase in the concentration of **PC** surfactants from 0.05% to 0.2% resulted in the continuous decrease in the linear swelling rate. At 0.2% of **PC** concentration, the linear swelling rate was reduced to 64.6% and 91.9% for 12 h and 24 h, respectively, compared to that of the linear swelling rate on bentonite pellets in the deionized water.

**Figure 7:**
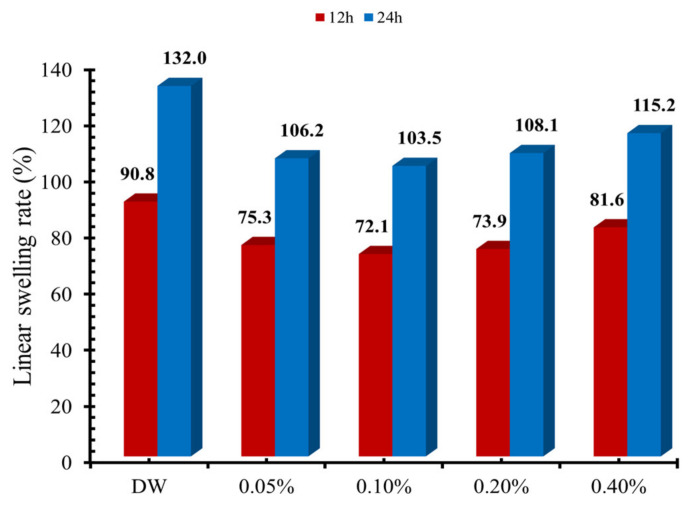
Effect of **PB** concentration and time on the linear swelling rate.

Nevertheless, a further increase in the concentration of **PC** increased the swelling. Figure 7 shows the effect of **PB** drilling mud formulation, concentration, and time on the linear swelling rate of bentonite pellets. The linear swelling rate of bentonite pellet was maximum in the deionized water, 90.8% for 12 h, and 132% for 24 h period, respectively. The addition of **PB** decreased the linear swelling rate using 0.05% and 0.1% concentration of **PB**. The minimum linear swelling rates observed were 72.1% and 103.5% at 0.1% concentration of **PB**, for 12-h and 24-h linear swelling experiments, respectively. However, the further increase in the concentration of **PB** caused a slight increase in the swelling rate. At lower concentrations, the adsorption of surfactants continued and resulted in a reduction of clay swelling. At higher concentrations, the adsorption reached a saturation state. Further increase in concentration adversely affected the inhibition performance, and as a result, the swelling rate increased. As reported by Hu et al., the surfactant concentration and surfactant arrangement in the layers of clay play a significant role in the swelling inhibition [80]. The alkyl chains take several arrangements, such as a lateral monolayer, a lateral bilayer, and a paraffin-type monolayer in the interlayer spacing of clay based on the surfactant concentration. At high concentration, the surfactant takes a paraffin-type monolayer arrangement exhibiting a larger tilting angle that leads to the maximum d-spacing of bentonite that adversely affects the inhibition performance. The alkyl arrangement of a particular surfactant requires detailed investigation as limited reports are available on this topic.

**Figure 8:**
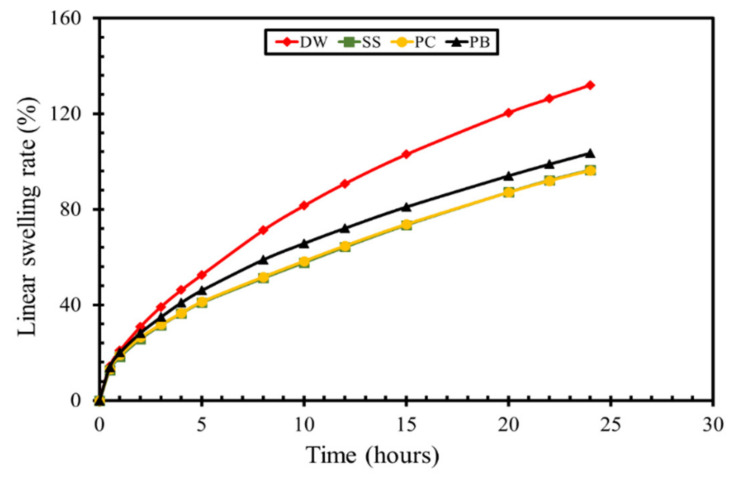
Comparison of linear swelling rate of various inhibitors.

Figure 8 shows the comparison of the linear swelling rate of bentonite pellets in the deionized water and modified drilling muds having a surfactant at 0.1% concentration for 24 h. It was observed that the linear swelling rate was maximum in the deionized water compared to all the drilling mud formulations. At the start of the experiment, the linear swelling rate of bentonite pellet was similar for all the formulation; however, after 5 h of experiment, a prominent distinction in linear swelling rate was observed for different drilling mud formulations. It was also found that the rate of swelling was abrupt in the initial phase of the experiment. The **PB** surfactant at 0.1% concentration showed a noticeable reduction in the linear swelling rate compared to the swelling rate in deionized water. The lowest rate was observed for the formulation having 0.1% sodium silicate and **PC**. The swelling rate of SS formulation and **PC** formulation was found to be approximately 97%, whereas the linear swelling rate for DW and **PB** was observed at 132% and 103%, respectively.

The impact of **PC** and **PB** on the linear swelling rate can be explained by the chemistry involved in the inhibition mechanism. The fluid providing the minimum linear swelling rate of bentonite pellets would be considered as superior for the inhibition of hydration and swelling of shale. The cationic ammonium ions in the drilling mud formulation chemically interact with the negatively charged clay through the electrostatic force of attraction, and the spacer group in the surfactant molecule acts as a hydrophobic layer on the clay surface. The long-chain alkyl groups of **PC** and **PB** act as hydrophobic groups that encapsulate the bentonite pellet keeping the water molecules away from the clay surface.

### 2.5. Capillary Suction Timer

**Figure 9:**
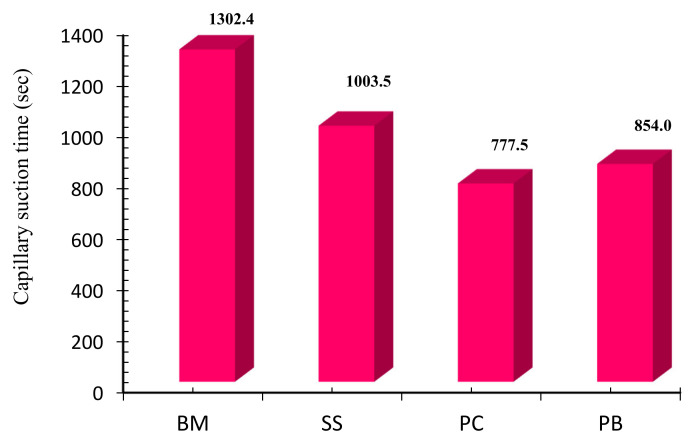
Capillary suction time test of base mud (BM), 0.1% of SS mud, and modified mud with 0.1% of **PC** and **PB**.

The capillary suction time (CST) test indicates the inhibition capacity of shale inhibitor used in the formulation of drilling mud. The magnitude of CST was measured in seconds for different drilling mud formulations with base mud (BM) and modified formulation with 0.1% concentration of shale inhibitor, as shown in Figure 9. The drilling mud formulation, which indicates a high magnitude of CST, mainly represents less inhibition capacity. Whereas, the drilling mud formulation that shows less magnitude of CST represents better inhibition capacity. In this study, the base mud (BM) shows the highest magnitude of CST, which is 1302.4 s, and the value of CST was attributed to less amount of free water available in the base mud. Whereas, the addition of 0.1% sodium silicate in the base mud results in the reduction of CST to 1003.4 s. The **PC**-modified formulations further reduced the CST to 777.5 s, and **PB**-based formulation reduced the CST to 854 s. The less CST time of surfactant-based drilling mud formulations shows that surfactant molecules replace the water present on the clay surface by inhibiting the clay, and that results in the free water that travels across the electrodes in the CST meter giving less CST time. The **PC**-modified formulation showed high inhibition performance as compared to other formulations.

### 2.6. Particle Size Distribution

**Figure 10:**
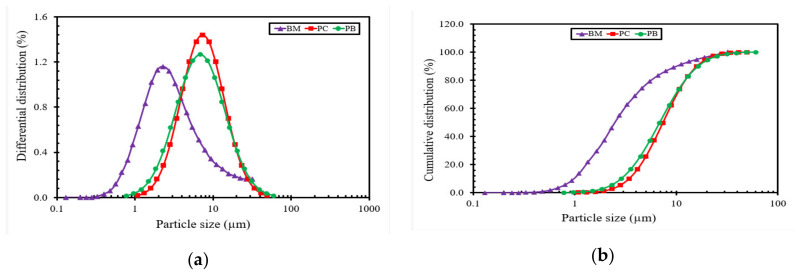
(**a**) Differential distribution of bentonite particle size in base mud and gemini surfactants (**PC** and **PB**)-modified muds; (**b**) Cumulative distribution of bentonite particle size in base mud and gemini surfactant (**PC** and **PB**) modified muds.

Particle size distribution test is one of the reliable techniques to analyze the inhibition characteristics of surfactants as shale inhibitors by measuring the average particle size distribution of clay. Bentonite swells and delaminates upon interaction with water, which leads to an increase in particles and a decrease in the average particle size of the hydrated bentonite [81]. The tendency of delamination depends on the swelling capability of montmorillonite mineral in bentonite [82]. Figure 10 shows the differential and cumulative distribution (%) of bentonite particle size in the base mud (BM) and gemini surfactants (**PC** and **PB**)-modified drilling mud formulations, respectively. It was observed that the addition of surfactants increased the size of the bentonite particles as the repulsive force among particle reduced and bentonite particles came closer to each other, resulting in increased size. The average particle size (d50) of bentonite in BM was about 2.7 µm, whereas **PC** formulation had a bentonite particle size approximately 7.4 µm. The **PB** drilling mud formulation showed a slight reduction in the average particle size compared to the **PC** formulation, as it had a bentonite particle size of 6.9 µm. The mean particle sizes were calculated from Figure 10b. The **PC**-based drilling mud formulation inhibited the swelling and hydration of bentonite particles; therefore, the average particle size of bentonite particles was bigger as compared to other formulations.

### 2.7. X-ray Diffraction

**Figure 11:**
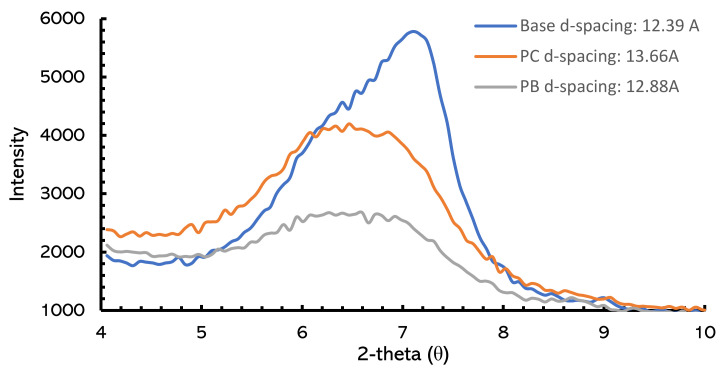
XRD patterns of bentonite hydrated in water and gemini surfactants (PC & PB).

In this study, the intercalation of gemini surfactants was investigated through the XRD technique, in which interlayer spacing or d-spacing was measured. The Interlayer or basal spacing(d) of bentonite changes upon ion-exchange reaction and informs us about the inhibition of clay. Figure 11 provides the XRD patterns of bentonite hydrated in water and gemini surfactants. It was observed that the addition of gemini surfactants increased the d-spacing of the interlayer, e.g., **PC** and **PB** surfactants increased the d-spacing from 12.39 Å to 13.66 Å and 12.88 Å, respectively. It is obvious that after the cation exchange, the basal spacing of bentonite moves toward lower angles, indicating the increase in interlayer spacing. This result confirms the intercalation of gemini surfactants in the interlayer, and it is in good agreement with the literature as well [79]. For an instant, Williams–Daryn [83] discussed the change in basal spacing upon the intercalation of bentonite by dicationic gemini surfactants. It was observed that gemini surfactants with larger cations with more amphiphilic properties created a hydrophobic region in the interlamellar layers of the clay and prevented hydration of clay.

## 3. Materials and Methods

### 3.1. Materials

The reagents used in this study are outlined in Table 2, with their purity and source. All reagents were used for the synthesis of gemini surfactants (**PC** and **PB**) without further purification. The details of other materials are also provided in Table 2.

### 3.2. Synthesis of **PC** and **PB**

**Figure 12:**
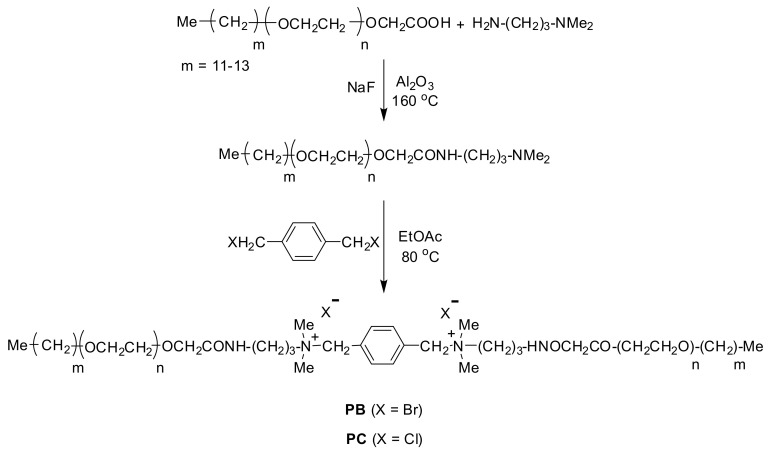
Synthetic outline of gemini surfactants (**PC** and **PB**).

Synthesis of gemini surfactants (**PC** and **PB**) was accomplished by mixing 3-(dimethylamino)-1-propylamine and glycolic acid ethoxylate lauryl ether, as shown in Figure 12. The reaction was carried out at 433 K for 10 h using sodium fluoride to accelerate the reaction, and an additional 3-(dimethylamino)-1-propylamine was added during the reaction for complete conversion of acid to an amide. A magnetic stirrer was used for a continuous stirring of the reaction mixture.

**Figure 13:**
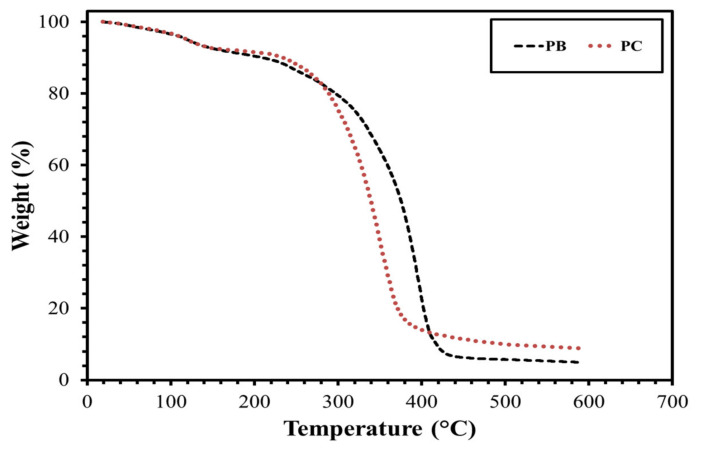
TGA analysis of **PC** and **PB**.

At the end of the chemical reaction, the extra amount of 3-(dimethylamino)-1-propylamine was removed under reduced pressure, and the crude was loaded in a glass column for column chromatography using EtOH as a mobile phase to obtain final product **PC** and **PB**. The confirmation of the chemical structure of the synthesized product was attained by proton and carbon NMR analysis. The detailed description of the synthetic protocol and spectral data analysis of **PC** and **PB** were described by Shakil et al. [84]. Besides, the thermogravimetric analysis (TGA) was performed on synthesized surfactants (**PC** & **PB**) to investigate their thermal stability as it is a prerequisite for their applications in oil and gas drilling operations (See, Figure 13). Both surfactants lost around 20% weight upon reaching 300 °C. After that, a sharp decline in weight loss of surfactants occurred, exhibiting the thermal degradation of surfactants. There was a minor impact of counterions on the thermal stability of surfactants. The **PB** surfactant containing bromide counterion showed better thermal stability as compared to **PC** containing chloride counterion.

### 3.3. Preparation of Drilling Muds

Drilling mud samples were prepared very carefully to achieve the desired properties. Three different classes of drilling mud were developed in this work. First, the base drilling mud (BM) was prepared by adding 6 wt.% bentonite to deionized water under constant stirring at 21,000 rpm using the Beach Hamilton mixer. Second, sodium silicate-based drilling mud (SS) was prepared by adding 6 wt.% bentonite to 0.1 wt.% stock solution of sodium silicate under constant high shearing. Third, the surfactant-based drilling muds (**PC** and **PB**) were prepared by mixing six wt.% bentonite to surfactant solutions having (0.1 wt.%) concentrations. All the drilling mud samples were prepared by applying vigorous stirring for 30 min. The sodium hydroxide solution (caustic soda solution of 14.5 pH) was added dropwise to all the drilling mud samples to adjust the pH to around 9. Each drilling mud sample was left at room temperature for 24 h before performing the test on drilling muds.

### 3.4. Rheological Measurements

Rheology of drilling muds is essential to determine the flow behavior under different shear rates for smooth drilling operations. The rheological properties, which are considered as critical for drilling muds, include apparent viscosity, plastic viscosity, yield stress, and gel strength. In this work, the rheological properties of various drilling mud formulations were determined by the M3600 automatic viscometer. The appropriate amount of drilling mud sample was charged to the viscometer cup, and pre-shearing was applied before performing the actual rheological measurements. The viscometer reading was taken at different rpm, as described by API recommendations, and standard Equations (1)–(3) were used to determine different rheological parameters. The gel strength of each drilling mud sample was determined at different time intervals as recommended by API standards, such as 10-s and 10-min gel strength. To determine the 10 s gel strength, the 10 s holding time was applied for each drilling mud, followed by the application of low shear at 3 rpm. Similarly, 10-min gel strength was measured by holding the drilling mud sample for 10 min, followed by the application of low shear at 3 rpm. The maximum deflection at 3 rpm was considered as the gel strength of the drilling muds. The following Bingham plastic model equations were used to determine different rheological properties:

(1) $$\mathrm{Apparent}\;\mathrm{viscosity}\;\left(\mu_{a}\right)\;=\;\frac{\emptyset_{600}}{2}\;(\mathrm{cp})$$

(2) $$\mathrm{Plastic}\;\mathrm{viscosity}\;\left(\mu_{p}\right)\;=\;\emptyset_{600}-\emptyset_{300}\;(\mathrm{cp})$$

(3) $$\mathrm{Yield}\;\mathrm{pint}\;\left(Y_{p}\right)\;=\;\emptyset_{300}-\mu_{p}\;\left(\frac{lb}{100ft^{2}}\right)$$

### 3.5. Filtration Test

The efficiency of drilling mud formulation can be determined by the amount of fluid loss and thickness of the filter cake as a result of the filtration process. In this study, the fluid loss characteristics were determined by the Fann filter press apparatus. To perform the filtration experiment, a Whatman filter paper no. 40 was placed at the bottom of the filter cup assembly and followed by the filling of a filter cup with 350 mL of drilling mud. The filter cup was tightly screwed, and the filtration experiment was started by applying 100-psi pressure using compressed air. The filtrate liquid was collected from the bottom of the filtration assembly after completing the filtration experiment for 30 min. At the end of the filtration experiment, the filter cake was carefully removed and washed with deionized water to remove excess drilling mud. The filter cake thickness was measured by Vernier caliper.

### 3.6. Linear Swelling Test

The linear swelling test was performed to assess the inhibition properties of prepared drilling mud at room temperature. The inhibition properties of drilling muds were determined on bentonite pellets. The bentonite pellet was made by taking 12 g of bentonite powder and compressed in the pellet making assembly by applying 6000 psi pressure for 30 min. The OFITE linear swelling meter was used for the standardized linear swelling tests. The prepared pellet was placed in the linear swelling cup and filled with 150 mL of made drilling mud along with a constant stirring of 100 rpm. The linear swelling rate of bentonite pellets was determined for 12 h and 24 h, at various concentrations of prepared drilling muds.

### 3.7. Capillary Suction Timer Test

The capillary suction time test was performed to characterize the effectiveness of drilling mud and the inhibition properties of the prepared drilling muds [85]. The capillary suction time test indicates the amount of free water available in the prepared drilling mud that will invade into the formation of the wellbore. The shale inhibitor in the drilling mud formulation interacts with the clay present in the drilling mud and releases the extra amount of water. In this study, several drilling mud formulations were made by mixing 0.1% concentration of different surfactants in the base mud and mixed thoroughly before performing the capillary suction time test. The prepared drilling mud was then added at the center of CST equipment, with a filter medium at the bottom of the drilling mud sample. The spreading rate of filtrate liquid is mainly affected by the chemical affinity of the inhibitor with drilling mud components and the nature of filter media used for the CST test. The drilling mud filtrate liquid moves outward in the radial direction, and the time in seconds was noted down for the filtrate liquid to reach the outer electrode of CST meter.

### 3.8. Wettability Alteration Measurements

Drop Shape Analyzer—DSA100 equipment was used to study the wettability alteration. The surfactant-based drilling muds were prepared with various concentrations, and each formulation was used to test the wettability of clay surface separately. A thin layer of each drilling mud formulation was spread on a glass plate and allowed to dry at room temperature for 24 h. Then, wettability of clay surface was determined by dropping a small droplet of deionized water, and the contact angle was measured with a resolution camera mounted on the equipment and the inbuilt software.

### 3.9. Particle Size Measurements

The drilling mud additives interact with each other, and as a result, the distribution of additives is altered. In this study, the effect of shale inhibitors (**PC** and **PB**) on the distribution of bentonite particles dispersed in deionized water was studied, and results were compared with base drilling mud with shale inhibitor. To measure the particle size distribution of bentonite, DT-1200 Acoustic Spectrometer was used. The surfactant-based drilling mud formulations were made by dispersing bentonite in the 0.1% solutions of **PC** and **PB** surfactants. All the formulations were thoroughly mixed using beach Hamilton mixer and left undisturbed before measuring particle size distribution.

### 3.10. X-ray Diffraction

The X-ray diffraction (XRD) analysis was conducted to investigate the interlayer or basal spacing (d) of bentonite hydrated in water and gemini surfactants. The XRD analysis was performed using the XRD system of PanAnalytical.

## 4. Conclusions

In this study, two different gemini surfactants (**PC** and **PB**) were synthesized, each having two quaternary ammonium cations, a benzene ring as a spacer, and two long alkyl chains along with negative counter ions (chloride or bromide). The shale inhibition performance of synthesized gemini surfactants was evaluated by several experimental techniques such as linear swelling test, particle size measurement, capillary suction time test, contact angle measurement, and XRD. Also, the impact of surfactant on rheology and fluid loss of muds was investigated.

- The addition of a surfactant in a base mud reduced its rheological parameters. The **PB** surfactant showed less impact on the rheology of base mud as compared to the **PC**.
- The fluid loss increased upon the addition of **PC** and **PB** in the base mud, which can be controlled by adding FLC.
- Both **PC** and **PB** changed the wettability of hydrated bentonite as indicated by contact angle measurement and made a hydrophobic surface.
- Reduction in CST time and increase in particle size depicted the clay swelling inhibition capacity of **PC** and **PB** gemini surfactants.
- The swelling rate of bentonite pellets was significantly reduced in the presence of **PC** and **PB** as compared to deionized water. **PC** formulation provided high inhibition same as sodium silicate in the linear swelling test.
- The gemini surfactants intercalated in the layers of bentonite and increased its basal spacing.
- The **PB** surfactant containing bromide counterion showed better thermal stability as compared to **PC** containing chloride counterion.

## Figures and Tables

**Table 1:** Rheological parameters (AV, PV & YP) of base mud (BM), 0.1% of SS mud and modified drilling muds with 0.1% of **PC** and **PB.**

Inhibitors	AV(cP)	PV(cP)	YP (l bf/100 ft^2^)
**BM**	11.1	8.2	5.7
**SS**	7.0	6.2	1.6
**PC**	7.4	5.6	3.7
**PB**	8.3	6	4.7

**Table 2:** List of reagents with their purity and source used in this study.

Name	Supplier	Purity (%)
Glycolic acid ethoxylate lauryl ether (690)	Sigma Aldrich	98.5
3-(dimethylamino)-1-propylamine	Sigma Aldrich	99
α,α’-dibromo-*p*-xylene	Sigma Aldrich	97
*α*,*α’*-dichloro-*p*-xylene	Sigma Aldrich	98
Aluminum oxide	Sigma Aldrich	99.99
Sodium fluoride	Sigma Aldrich	99
Sodium hydroxide	Sigma Aldrich	98
Sodium Bentonite	Halliburton	-
Sodium silicate	Sigma Aldrich	-
